# Monthly Summary

**Published:** 1875-06

**Authors:** 


### MONTHLY SUMMARY.

---

*Treatment of Cold.*—The editor of the *Archives of Electrology and Neurology*, in the number for March, 1874, says :—

We have long been in the habit of using what we call a "cold powder," which we have found of great value in breaking up colds when taken in time, and in modifying their force when taken late. The prescription is as follows ;—Dissolve five parts of camphor, in ether, to the consistence of cream ; then add carbonate of ammonia four parts ; opium-powder, one part. Mix and keep in a tightly corked bottle. The dose is regulated by the opium, and ranges between three and ten or fifteen grains. We have been accustomed to prescribe it for our friends by the finger-nail full, or as much as one can put on a finger-nail. This powder may be taken in a little water just before retiring, by preference, or at any hour of the day, whenever there is a suspicion of having caught cold. If need be, a moderate dose may be taken several days in succession.

The advantages of this powder are very great. 1. The taste is agreeable, or at least it is not disagreeable. Even the bitterness of the opium is mostly neutralized by the camphor and ammonia. No child objects to this powder. 2. It is singularly and inexplicably efficacious. We believe it to be more efficient than Dover's powder, and incomparably more agreeable. In some cases it produces a gentle perspiration ; in others, this especial effect is not observed. It is so easy to take, and so harmless in small doses, that it is well and safe to take it whenever we become badly chilled. We first call attention to this cold powder in 1869. From various sources, lay and medical, we hear that it accomplishes all that is here asserted, and we therefore earnestly recommend it to the profession.—*Med. and Surg. Reporter.*

---

*Nelaton's Method of Resuscitation from Chloroform Narcosis*—Dr. I. Marion Sims, [*Brit. Med. Jour.*,] most impressively details his observations respecting the practical workings of the above method. Briefly stated, it consists of in reversing the body of the patient, either by hanging him up by the feet or laying him over a bed or table, so that the blood from the greater part of the body can gravitate towards the head. The tongue is drawn forward by a tenaculum and artificial respiration tried. This practic is based on the theory that death from chloroform takes place from cerebral anæmia.—*Detroit Review of Medicine and Pharmacy.*

---

*An Antidote to Chloroform.*—Dr. Schuller has discovered that the nitrite of amyl quickly removes the effects of chloroform on the vessels of the pia mater, and that even in cases of advanced narcotism from the latter drug it rapidly relieves the dyspnœa and labored respiration, restoring the strength of the pulse, and the reflex excitability. This discovery may prove of much practical value where chloroform contnues to be the favorite anæsthetic.—*New York Med. Journal,*

*Solvents for Rubber.*—For the information of correspondents, several of whom have made inquiries on the above subject, we give the following:

The proper solvents for caoutchouc are ether, [free from alcohol,] chloroform, bisulphide of carbon, coal naphtha, and rectiffied oil of turpentine. By long boiling in water, rubber sotens, swells, and becomes more soluble in its peculiar menstrua; but when exposed to the air it speedily resumes its pristine consistence and volume. Industrially, the ethereal solution of caoutchouc is useless, because it contains hardly more than a trace of that substance. Oil of turpentine dissolves caoutchouc only when the oil is very pure, and with the application of heat; the ordinary oil of turpentine of commerce causes india-rubber to swell rather than to become dissolved. In order to prevent the vicosity of the india-rubber when evaporated from its solution, one part of caoutchouc is worked with two parts of turpentine into a thin paste, to which is added one-half part of a hot concentrated solution of sulphuret of potassium in water; the yellow liquid formed leaves the caoutchouc perfectly elastic and without any vicosity. The solutions of caoutchouc in coal-tar naphtha and benzoline are most suited to unite pieces of caoutchouc, but the order of the solvents is perceptible for a long time. As chloroform is too expensive for common use, sulphide of carbon is the most usual, and also the best solvent for caoutchouc. This solution, owing to the volatility of the menstrum, soon dries, leaving the latter in its natural state. When alcohol is mixed with sulphide of carbon, the latter does not any longer dissolve the caoutchouc, but simply softens it and renders it capable of being more readily vulcanized. Alcohol also precipitates solutions of caoutchouc. When caoutchouc is treated with hot naphtha distilled from native petroleum or coal-tar, it swells to thirty times its former bulk; and if then triturated with a pestle and pressed through a sieve, it affords a homogeneous varnish, the same that is used in preparing the waterproof cloth of Mackintosh. Caoutchouc dissolves in the fixed oils, such as linseed oil, but the varnish has not the property of becoming concrete on exposure to the air. Caoutchouc melts at a head of about 256° or 260°; after it has been melted it does not solidify on cooling, but forms a sticky mass, which does not become solid, even when exposed to the air for months. Owing to this property, it furnishes a valuable material for the lubrication of stopcocks and joints intended to remain air-tight, and yet be movable.—*Druggists' Circular.*

---

*Singular Death.*—Dr. Wm. E. Rossiter, of Bridgeport, Conn., has just died from the effects of inhaling ether, which he was administering to a patient four weeks ago. It produced symptoms similar to those of typhoid fever and diphtheria.

*Death after Intra-venous Injection of Chloral.*—The success which has attended a small series of some twenty cases of intra-venous injection of chloral seems to have rendered some practitioners overbold, and the worst is that they do not always perceive the cause of fatal results when these have been produced. In a note to Prof. Ore, of Bordeaux, the originator of this practice, Dr Lande relates a case of ovariotomy in which sleep was induced in the space of thirteen minutes, after twenty five grammes of an aqueous solution of chloral, containing five grammes of chloral, had been injected. The insensibility produced was absolute, and the operation, owing to adhesions which had to be ruptured with the hand, lasted about half an hour. The patient soon began to sink, and she died in little more than an hour after the operation had been commenced. There was a moderate amount of hemorrhage, which, in the anæmic condition of the patient, the reporter considers sufficient to account for death. Such a conclusion, however, M Jeannel observes, reporting on the case in the *Union Medicale*, will certainly be disputed, since it is far more probable that death arose from the influence of the anæsthetic directly introduced into the circulation, the effects of which could be neither modified nor arrested. " The whole therapeutical history of chloral," he adds, " should teach us to employ it with the greatest caution. If intra-venous injections of this redoubtable agent can be justified up to a certain point for the treatment of diseases against which medicine up to the present time remains unarmed, such as tetanus and hydrophobia, we refuse absolutely to admit that these injections should replace inhalations of chloroform, the effects of which can be prudently controlled, increased, or interrupted according to the indications furnished by the great organic functions."—*Med. Times and Gazette.*

---

*Intravenous Injection of Chloral for Producing Anæsthesia.*—At a late meeting of the Paris Academy of Sciences, M. Ore forwarded particulars of two fresh cases of anæsthesia, produced by the intravenous injection of chloral. In one case the object proposed was to scoop out the tibia on account of caries of the bone, the other was for the operation of ovariotomy. Anæsthesia in both cases was complete, and was neither accompanied nor followed by any accident which could be attributed to the chloral. M. Ore took the opportunity to point out the means of neutralizing the possible acidity of the chloral, a circumstance which might possibly bring on coagulation of the blood in the veins. For this purpose it is sufficient to dissolve one gramme of carbonate of soda in ten grammes of distilled water, and to add two or three drops of this solution to a solution of one gramme of chloral in four of water.—*London Med. Record.*

